# The Relation of Drugs to Pregnancy

**Published:** 1890-11

**Authors:** L. Hamilton Evans

**Affiliations:** Toronto, Canada; Bureau of Obstetrics, I. H. A.


### THE RELATION OF DRUGS TO PREGNANCY.

L. Hamilton Evans, B. A., M. D., Toronto, Canada.

(Bureau of Obstetrics, I. H. A.)

"I have got in the family way, and I want you to give me something to get me all right."

Such is the statement, such the request occasionally made to us by some of our patients. I cannot say what answers you are accustomed to give, probably they are various, according to the varying circumstances of individual cases, and your own peculiarities of thought and language. The following reply would, in most cases, perhaps, be substantially appropriate. "If you are right, you are wrong, because you are all right already. In other words, if you are correct in stating that you are in the family way, you are mistaken in supposing that you require medicine to cure you of that condition, because it is not, of itself, a disease."

This, I repeat, would be in many cases, a proper reply, though not always an acceptable one to our applicant. Disease might be roughly defined to be a state or condition of a living organism in which the never-ending changes which constitute life are not succeeding each other in proper order—are not going on in such a manner as to conduce to the physical aim or object of its material existence—that this aim or object is two-fold. First, the comfort or pleasure of the living creature; second, the reproduction of its kind—the perpetuation of its species. Now, if these two objects are associated in one individual by a perfect intelligence, they cannot be incompatible with each other. Consequently the carrying out of the instinct of reproduction, in the case of beings in a perfect state of nature, is not incompatible with the enjoyment of a condition of perfect comfort; in other words, pregnancy is not, in itself, a disease.

This being the case, and seeing we use drugs, as such, for the cure of disease, how can there exist any relation between drugs and pregnancy? It would seem, at first sight, that the whole subject of this paper might be disposed of in as few words as the celebrated chapter on the snakes of Ireland. On more careful consideration, however, we find that it presents a few points worthy of being studied.

The fact is, the whole human race, or at least that portion of it with which we have directly to do, is born in a state of hereditary disease, dormant, it is true, in many cases, yet none the less a source of danger and distress to come on this account; perhaps, indeed, more dangerous because latent, as a foe in ambush is more to be dreaded than one arrayed against us in the open field of battle. As age advances, and the various functions of nutrition and reproduction successively develop, the increased activity of the vital forces, acting upon and being acted upon by this development, is accompanied by an increased activity in the latent germs of disease, and necessarily so, since we must suppose that the force which develops one phase of vitality will develop another, and disease itself is life only gone astray.

In illustration of these remarks, I may refer briefly to the period of dentition in infants. If teeth are necessary adjuncts of nutrition in certain stages of our existence, and if we come into the world without them it cannot be that we must necessarily undergo suffering in acquiring them. Yet, as a matter of fact, we do often find ourselves compelled to endure much pain and discomfort in the act. It is quite common to connect the two together as bearing the direct relation of cause and effect. But this is proved to be a mistake, for the reason already given. Whence, then, the suffering so often observed? It can only be the result of a previously existing and possibly up to this time latent condition of disease, and on this ground alone can we attempt to relieve the pain, with radical benefit to the sufferer. And here it is worth remarking that if we treat such cases, and all cases of suffering, with due regard to the underlying disorder, we not only avoid the damage which the use of mere palliatives would inflict, but we confer a lasting benefit upon the sufferer.

The foregoing remarks will suggest a reason why we may frequently find the use of drugs applicable to the condition of pregnancy. I should rather say applicable to a patient in that condition. This state not only exalts the vital functions of the prospective mother to the highest point in themselves, but it appears to go a step further. The new being not only receives life from the parent, but by the law of reaction imparts vitality in its turn. I can only in this way account for the fact that some women enjoy better health at this time than at any other, some cases of consumption even being to all appearance temporarily stayed. It is true many suffer severely while in this condition, and for a somewhat similar reason as before explained.

Now, seeing that the action of drugs in the cure of disease is indirect, and depends upon the reaction of the vital forces; seeing, furthermore, that many cases of apparently acute disease are not radically acute, but only the acute manifestation of a chronic cachexia; it follows that if we have at heart the permanent benefit of our patients, we should ever be on the watch for such occasions of treating acute disorders, as well as chronic ailments, as are presented to us when the vitality of our patients is at a high point of activity. Such an occasion is offered, perhaps more than under any other circumstances, by the condition of pregnancy.

